# Comparative Study Between the Effects of High Doses of Rosuvastatin and Atorvastatin on Ventricular Remodeling in Patients with ST-Segment Elevation Myocardial Infarction

**DOI:** 10.1007/s10557-024-07621-w

**Published:** 2024-09-12

**Authors:** Zeinab M. Elhadad, Amira B. Kassem, Ahmed Mahmoud El Amrawy, Ahmad Salahuddin, Noha A. El-Bassiouny

**Affiliations:** 1 Department of Clinical Pharmacy and Pharmacy Practice, Faculty of Pharmacy, Damanhour University, Damanhour City, Egypt; 2 Department of Cardiology, Faculty of Medicine, Alexandria University, Alexandria City, Egypt; 3 Department of Biochemistry, Faculty of Pharmacy, Damanhour University, Damanhour City, Egypt; 4 Department of Biochemistry, College of Pharmacy, Al-Ayen Iraqi University, Thi-Qar, 64001 Iraq

**Keywords:** High dose atorvastatin, High dose rosuvastatin, Ventricular remodeling, Cardiac function, MMP9, solubleST2

## Abstract

**Background:**

Most studies reported that treating ST-Elevation Myocardial Infarction (STEMI) patients with high doses of rosuvastatin or atorvastatin could improve left ventricular remodeling and cardiac function.

**Purpose:**

The current study compared the impact of high doses of rosuvastatin and atorvastatin on hypertrophy, fibrosis markers, serum inflammatory markers, and left ventricular function in STEMI patients after primary percutaneous coronary intervention (PCI).

**Method:**

After primary PCI, eighty STEMI patients were randomized to receive either 20 mg of rosuvastatin (*n* = 40) or 40 mg of atorvastatin (*n* = 40) once daily for 3 months. Soluble Suppression of Tumorigenicity-2 (sST2), Matrix Metalloproteinase-9 (MMP9), C-Reactive Protein (CRP), lipid parameters, liver enzymes, and echocardiographic parameters were assessed for the two groups at baseline and after 3 months.

**Results:**

After 3 months of treatment, a statistically significant reduction was observed in the rosuvastatin group regarding the levels of CRP (16 ± 6 vs. 20 ± 10 mg/L, *P* = 0.024) and MMP9 (104 ± 33 vs. 130 ± 42 ng/L, *P* = 0.003) compared with the atorvastatin group. The median percentage decrease in sST2 level in the rosuvastatin group was higher (6.1%) than in the atorvastatin group (2.3%) after 3 months of treatment. Also, in the rosuvastatin group, LVEF was significantly increased (48.5 ± 9 vs. 43.5 ± 11%, *P* = 0.029), while LVEDV and LVESV were significantly decreased compared to those of the atorvastatin group (101 [81/135] vs. 134 [100/150] ml, *P* = 0.041) (53 [37/75] vs. 73 [52/92] ml, *P* = 0.033), respectively.

**Conclusion:**

High-intensity rosuvastatin was superior to high-intensity atorvastatin in reducing the inflammatory response and myocardial fibrosis, thus improving ventricular remodeling and cardiac function better in STEMI patients.

**Trial Registration:**

This randomized controlled trial was registered on October 11, 2022, on ClinicalTrials.gov under registration number: NCT05895123 “retrospectively registered”.

**Supplementary Information:**

The online version contains supplementary material available at 10.1007/s10557-024-07621-w.

## Introduction

Coronary artery disease (CAD) is the most prevalent cause of mortality from cardiovascular diseases (CVD) worldwide [1, 2]. Acute coronary syndrome (ACS) is the acute manifestation of CAD [2]. A serious form of ACS is ST-Elevation Myocardial Infarction (STEMI), which happens when an atherosclerotic plaque ruptures, followed by a complete thrombotic occlusion in the coronary circulation, leading to myocardial ischemia and injury [2–4]. This injury triggers adaptive immunological responses, which cause the heart to remodel its structure and function to preserve the cardiac outcome; these changes are known as left ventricular remodeling (LVR) [5]. During its early phase, LVR occurs as a result of myocardial necrosis and inflammation, which break down the collagen scaffolding, resulting in ventricular shape alteration, regional thinning, and infarcted myocardium dilatation [2, 5, 6], while in the late phase of LVR, cardiac fibrosis, left ventricular dilatation, and hypertrophy occur [5, 6]. As a result, there is a progressive increase in LV volume and a decrease in LV ejection fraction (LVEF), ultimately leading to chronic heart failure, a significant cause of morbidity and death among patients with MI [5, 7]. Furthermore, LVR is essential in evaluating prognosis and cardiac function in MI patients. Hence, preventing or treating myocardial fibrosis and reversing or stopping ventricular remodeling will enhance the beneficial outcomes [8, 9].

Owing to the intricate nature of remodeling’s pathophysiology, it may be beneficial for remodeling prediction to combine biomarkers from various groups that reflect different remodeling pathways [10, 11]. Inflammatory biomarkers such as C-Reactive Protein (CRP), myocardial fibrosis, and hypertrophy biomarkers such as Matrix Metalloproteinase-9 (MMP9) and soluble Suppression of Tumorigenicity 2 (sST2) have been introduced as promising biomarkers for LVR prediction [6, 11]. CRP is an acute-phase protein produced by the liver. During acute myocardial infarction (AMI), interleukin-6 produced from the infarct area stimulates CRP production, so myocardial ischemia is associated with systemic inflammatory response [10]. MMPs are extracellular matrix (ECM) protein-degrading enzymes that belong to the zinc-dependent endopeptidases family [10]. Cardiomyocytes are surrounded by ECM proteins, which maintain the LV geometry and shape [10]. MMP9 expression increases in AMI patients under hypoxic conditions, resulting in accelerated collagen degradation, myocardial fibrosis, compensatory ventricular dilation, and ventricular remodeling [12]. Thus, it is important to predict LVR and the patient’s prognosis [13]. ST2 is a member of the IL-1 receptor family and has two major isoforms: the ST2 transmembrane receptor ligand (ST2L) and the soluble receptor ST2 (sST2), which circulates in the bloodstream. ST2 is secreted by fibroblasts and cardiomyocytes subjected to stimuli such as inflammation, fibrosis, or stress [14]. ST2L and IL-33 binding on the membrane of cardiomyocytes protect against the adverse remodeling effects of Angiotensin II, preventing cardiac fibrosis and hypertrophy [6]. During AMI, sST2 levels increase and prevent ST2L and IL-33 binding, attenuating their cardioprotective effects because sST2 binds with IL-33 and reduces its availability to binding with ST2L. Therefore, elevated sST2 secretion after MI is associated with increased cardiac fibrosis, LVR, post-MI heart failure, and prediction of mortality [6, 10].

Guidelines currently recommend high-intensity statin therapy for the management of STEMI patients because it reduces morbidity and mortality more efficiently [15, 16]. The significant reduction in plasma cholesterol produced by high-intensity statin stabilizes atherosclerotic plaque, thus reducing inflammation [17, 18]. Moreover, clinical studies have shown that statins also modulate ventricular remodeling by inhibiting neurohumoral activation; reducing inflammation; influencing the signals that lead to fibroblast growth, myocyte hypertrophy, and myocyte loss; and inhibiting MMPs [18–21]. Statins inhibited the MMP9 secretion process with no effect on MMP9 activity due to inhibition of macrophage activation, thus further improving in the patient prognosis [13, 22].

Rosuvastatin and atorvastatin are the two most widely prescribed statins in clinical settings [23]. Different clinical studies reported that treating STEMI patients with high doses of rosuvastatin or atorvastatin could improve LVR and cardiac function [9, 24–27]. However, there is a paucity of head-to-head clinical studies comparing high doses of rosuvastatin and atorvastatin regarding LVR and left ventricle function in STEMI patients after primary percutaneous coronary intervention (PCI). Thus, the primary objective of this study was to compare the impact of high doses of rosuvastatin and atorvastatin on myocardial fibrosis, hypertrophy biomarkers, serum inflammatory biomarkers, and left ventricular function in STEMI patients.

## Methods

### Study Design

This study was a single-blind, randomized, parallel, comparative study carried out at Alexandria Main University Hospital (AMUH) cardiology department. This study followed the ethical standards of the 1964 Declaration of Helsinki. Ethical approvals were obtained from the Ethics Committee of Damanhur University (Reference No. 921PP38) and Alexandria University (Reference No. 0106987). Written informed consent was signed for all patients. The trial was registered on clinicaltrials.gov with the ID NCT05895123 (https://clinicaltrials.gov/study/NCT05895123).

### Patients and Drug Intervention

Between November 2021 and April 2023, two hundred thirty-eight STEMI patients were enrolled, and eighty-nine of them fulfilled the inclusion and exclusion criteria. Using a computer-generated randomization system, patients were randomized equally in a 1:1 ratio to receive either 20 mg of rosuvastatin or 40 mg of atorvastatin for 3 months. Finally, only 80 patients completed the follow-up period, with 40 cases in the atorvastatin group and 40 cases in the rosuvastatin group. According to the European Society of Cardiology (ESC) guidelines, the diagnosis of STEMI was confirmed [3]. According to ESC guidelines, both groups received Angiotensin-Converting Enzyme Inhibitor (ACE-I), anticoagulant, dual antiplatelets (Aspirin and Ticagrelor or Clopidogrel), and β receptor blocker, additionally to statin treatment [15].

### Inclusion and Exclusion Criteria

Patients with the following criteria were included: abnormal ST segment elevation in an electrocardiogram and fulfilled the criteria of STEMI diagnosis [3]; patient suffered a first myocardial infarction; successful one-stage primary PCI therapy was administered within 12 h. The patients with the following criteria were excluded: severe cardiac insufficiency; either liver or kidney insufficiency; use of antioxidant or other antilipidemic drugs during the first 2 weeks of hospitalization; history of severe allergy, familial hypercholesterolemia; immune system diseases; malignant tumors; acute infectious diseases; serious blood system diseases; and contraindication to rosuvastatin and atorvastatin.

### Study Procedures and Biomarker Measurement

After a 10-h of overnight fasting, venous blood from all patients was withdrawn at baseline and after 3 months. At baseline, blood electrolytes, blood urea nitrogen, and serum creatinine were measured. Moreover, serum levels of total cholesterol (TC), high-density lipoprotein cholesterol (HDL-C), low-density lipoprotein cholesterol (LDL-C), triglyceride (TG), alanine transaminase (ALT), aspartate transaminase (AST), and CRP were measured at baseline and after 3 months by spinlab semi-automated biochemical analyzer. For measurement of MMP9 and sST2 at baseline and after 3 months, serum was separated and aliquoted after blood samples centrifugation for 20 min and frozen at − 80 °C to be stored until the assay time. Both sST2 and MMP9 levels were assessed by enzyme-linked immunosorbent assay (ELISA). sST2 was measured using the human (sST2/sIL 33 R/IL-1Srl1) ELISA kit (Sunred Biological Technology Co., Ltd., Shanghai, China). Also, MMP9 was measured using the human MMP9 ELISA kit (Sunred Biological Technology Co., Ltd., Shanghai, China). Lab technicians, nurses, and anyone who collected blood samples and performed laboratory analysis were blinded to the group assignment.

### Cardiac Echocardiography

At baseline and after treatment with statin for 3 months, transthoracic echocardiography was performed. All patients were examined using a machine (PHILIPS, EPIC 7C) in the left lateral position. Comprehensive transthoracic M-mode, 2D, and Doppler were performed in standard views. LV end-diastolic volume (LVEDV) and LV end-systolic volume (LVESV) were derived from apical 4 and 2 chamber views. LVEF was determined using Simpson’s biplane rule. The echocardiography was performed for all patients by the same independent cardiologist who was blinded to the treatment allocation to exclude any possible bias.

### Follow-up and Adherence

A follow-up visit to the cardiac clinic was scheduled once a month for 3 months to ensure patient adherence, record any serious cardiac events, and monitor drug adverse effects. After 3 months, all the patients were assessed for elevated liver enzymes (ALT and AST) associated with statin. Also, they were asked if they experienced any of the most common adverse effects of statins, such as myalgia, headache, asthenia, diarrhea, dyspepsia, nausea, and constipation [28, 29]. The pill count method was used to ensure the patient’s adherence to their treatment.

### Sample Size Calculation

Using NCSS 2004 and PASS 2000 programs, the sample size was calculated based on published data from a previous study (El-Said NO et al., 2020) [30]. A minimum required sample size of 40 patients in each group is needed to achieve 80% power and detect a 5 (pg/ml) difference in sST2 between both groups using a two-sided independent sample *t*-test at a significance level of 0.05.

### Statistical Analysis

IBM SPSS statistics software version 24 was used for statistical data analysis. The Kolmogorov–Smirnov test was used to determine if the distribution of the data was normal. Normally distributed data were presented as mean ± standard deviations and compared using a paired *t*-test within the same group (at baseline vs. after 3 months of treatment) and an unpaired *t*-test between the two groups. Non-normally distributed data were presented as median with interquartile range (25th/75th percentile) and compared using the Wilcoxon signed-rank test within the same group (at baseline vs. after 3 months of treatment) and the Mann–Whitney *U* test between the two groups. Categorical variables were expressed as numbers (percentage) and compared using the chi-square (X2) test between the two groups. Correlation between variables was performed using Pearson correlation. The significance level for all tests was set up at *P* < 0.05.

## Results

### Baseline Characteristics

**Fig. 1:**
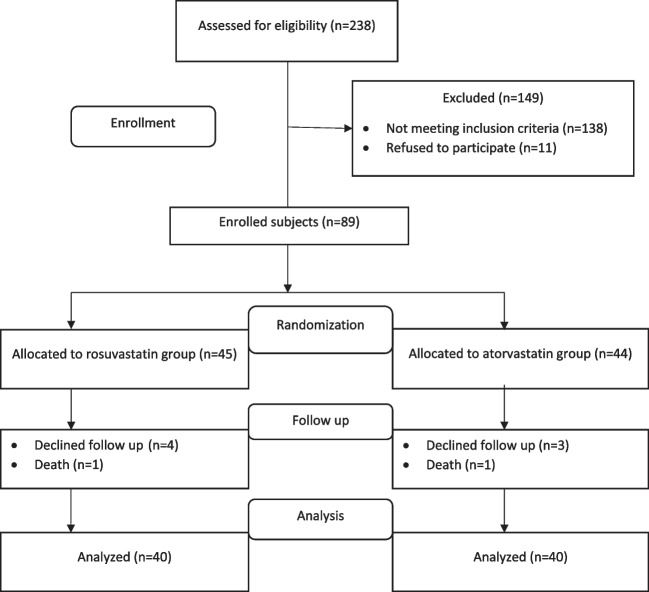
Flow chart for participants’ screening, randomization, follow-up, and analysis

At first, two hundred thirty-eight STEMI patients were enrolled; 138 patients were not eligible for inclusion, 11 refused to participate, and only 89 were initially included in this study. After randomization, eighty-nine patients were allocated into the rosuvastatin group (*n* = 45) and the atorvastatin group (*n* = 44). During the 3-month follow-up period, in the rosuvastatin group, four patients declined follow-up attendance, and one patient died, while in the atorvastatin group, three patients declined follow-up attendance, and one patient died. Finally, forty patients completed this study in each group, as illustrated in Fig. 1. At baseline, there was no statistically significant difference in age, gender, risk factors, BMI, kidney functions, liver functions, other laboratory parameters, cardiac injury and necrosis biomarkers (creatine kinase-myocardial band and troponin-hs), onset-to-balloon time, culprit lesion, and co-administered medications between both groups **(**Table 1**)**. The revascularized vessel in both atorvastatin and rosuvastatin groups was single vessel-culprit only in all patients.

**Table 1:** Baseline characteristics of patients in both groups before treatment

Parameter	Atorvastatin group (*n* = 40)	Rosuvastatin group (*n* = 40)	*P* value
Age (years)	53 ± 10	56 ± 10	0.290
Gender *n* (%)			
Male Female	35 (87.5%) 5 (12.5%)	32 (80%) 8 (20%)	0.714 0.405
BMI (kg/m^2^)	26 (25/27)	27 (25/28)	0.358
Risk factor			
Hypertension *n* (%) Diabetes mellitus *n* (%) Smoking *n* (%)	11 (27.5%) 13 (32.5%) 34 (85%)	12 (30%) 12 (30%) 30 (75%)	0.835 0.842 0.617
Heart rate (beats/min)	87 ± 18	81 ± 13	0.139
Systolic blood pressure (mmHg)	80 (73/98)	80 (70/88)	0.12
Diastolic blood pressure (mmHg)	130 (110/140)	125 (110/140)	0.883
Laboratory tests			
ALT (U/L) AST(U/L) TG (mg/dl) TC (mg/dl) LDL-C (mg/dl) HDL-C (mg/dl) Serum creatinine (mg/dl) Blood urea (mg/dl) BUN (mg/dl) Serum sodium (mmol/l) Serum potassium (mmol/l)	56 ± 24 54 ± 19 129 (105/202) 214 ± 54 136 ± 54 47 ± 12 1.01 ± 0.22 25 (20/34) 12 (12/16) 136 ± 4 4.22 ± 0.49	50 ± 16 55 ± 18 135 (104/229) 202 ± 55 125 ± 58 46 ± 16 1.03 ± 0.26 27 (21/34) 12.5 (12.5/16) 136 ± 5 4.1 ± 0.43	0.206 0.793 0.799 0.369 0.385 0.543 0.675 0.636 0.828 0.895 0.277
Cardiac injury and necrosis biomarkers			
CK-MB (ng/ml) Troponin-hs (ng/ml)	82 (13/215) 24 (8/50)	119 (9/203) 25 (8/38)	0.855 0.965
Culprit lesion *n* (%)			
RCA LAD LCx	10 (25%) 28 (70%) 2 (5%)	13 (32.5%) 26 (65%) 1 (2.5%)	0.532 0.785 0.564
Onset-to-balloon time (hour)	5.8 ± 1.8	6.15 ± 1.7	0.411
Co-administrated medications *n* (%)			
ACEIs (ramipril) Beta-blockers (bisoprolol) Aspirin P2Y12 inhibitor (ticagrelor) P2Y12 inhibitor (clopidogrel) Spironolactone PPI (pantoprazole) Loop diuretics (torsemide) Anticoagulant (rivaroxaban) SGLT-2 inhibitor (empagliflozin)	30 (75%) 26 (65%) 40 (100%) 31 (77.5%) 9 (22.5%) 5 (12.5%) 40 (100%) 4 (10%) 4 (10%) 8 (20%)	31 (77.5%) 35 (87.5%) 40 (100%) 33 (82.5%) 7 (17.5%) 7 (17.5%) 40 (100%) 3 (7.5%) 3 (7.5%) 14 (35%)	0.898 0.249 1 0.803 0.617 0.564 1 0.705 0.705 0.201

*BMI*, body mass index; *TG*, triglyceride; *TC*, total cholesterol; *LDL-C*, low-density lipoprotein cholesterol; *HDL-C*, high-density lipoprotein cholesterol; *ALT*, alanine transaminase; *AST*, aspartate transaminase; *RBS*, random blood sugar; *BUN*, blood urea nitrogen; *CK-MB*, creatine kinase-myocardial band; *RCA*, right coronary artery; *LAD*, left anterior descending artery; *LCx*, circumflex artery; *ACEI*, angiotensin-converting enzyme inhibitors; *P2Y12*, purinergic receptor type Y, subtype 12; *PPI*, proton pump inhibitor; *SGLT-2*, sodium-glucose co-transporter. Data are represented as mean ± SD for quantitative normally distributed data, median (25th/75th percentile) for quantitative non-normally distributed data. Unpaired t-test, Mann–Whitney test as appropriate statistically significant between two groups at *P* < 0.05

### Effect of Statins on Lipid Parameters

Both atorvastatin and rosuvastatin groups showed a significant reduction in TC, LDL-C, and TG levels and a significant elevation in HDL-C levels after 3 months of treatment compared to their corresponding baseline values (*P* < 0.05). However, after 3 months of treatment with atorvastatin and rosuvastatin, the difference between the two groups was insignificant **(**Table 2**)**.

**Table 2:** Effect of high dose atorvastatin and rosuvastatin on liver enzymes and lipid profiles at baseline and after treatment

Parameters		Atorvastatin group (*n* = 40)	Rosuvastatin group (*n* = 40)	*P*-value
TG (mg/dl)	Baseline	129 (105/202)	135 (104/229)	0.799
	After 3 months	103 (73/135)	106 (62/127)	0.353
	*P*-value	0.001^b^	< 0.001^b^	
TC (mg/dl)	Baseline	214 ± 54	202 ± 55	0.369
	After 3 months	166 ± 52	160 ± 54	0.639
	*P*-value	< 0.001^b^	< 0.001^b^	
LDL-C (mg/dl)	Baseline	136 ± 54	125 ± 58	0.385
	After 3 months	91 ± 52	88 ± 49	0.794
	*P*-value	< 0.001 ^b^	0.001^b^	
HDL-C (mg/dl)	Baseline	47 ± 12	46 ± 16	0.543
	After 3 months	52 ± 10	51 ± 9	0.482
	*P*-value	0.028^b^	0.039^b^	
ALT (U/L)	Baseline	56 ± 24	50 ± 16	0.206
	After 3 months	21 ± 7	17 ± 5	0.004^a^
	*P*-value	< 0.001^b^	< 0.001^b^	
AST (U/L)	Baseline	54 ± 19	55 ± 18	0.793
	After 3 months	24 ± 7	24 ± 7	0.827
	*P*-value	< 0.001^b^	< 0.001^b^	

*TG*, triglyceride; *TC*, total cholesterol; *LDL-C*, low density lipoprotein cholesterol; *HDL-C*, high density lipoprotein cholesterol; *ALT*, alanine transaminase; *AST*, aspartate transaminase. Data are represented as mean ± SD for quantitative normally distributed data median (25th/75th percentile) for quantitative non-normally distributed data. ^a^Statistically significant difference between the two groups. ^b^Statistically significant difference between baseline and after treatment in each group. Unpaired *t*-test, paired *t*-test, Mann–Whitney test, and Wilcoxon signed ranks test, as appropriate statistically significant difference at *P* < 0.05

### Effect of Statins on Myocardial Fibrosis, Hypertrophy, and Serum Inflammatory Biomarkers

In both atorvastatin and rosuvastatin groups, a statistically significant reduction in the levels of CRP and MMP9 was observed after treatment compared with their corresponding baseline values (*P* < 0.05). In addition, the levels of CRP (16 ± 6 vs. 20 ± 10 mg/L, *P* < 0.05) and MMP9 (104 ± 33 vs. 130 ± 42 ng/L, *P* < 0.05) were significantly decreased in the rosuvastatin group compared to the atorvastatin group after three months of treatment **(**Table 3**)**.

**Table 3:** Effect of high dose atorvastatin and rosuvastatin on CRP and MMP9 at baseline and after treatment

Parameters		Atorvastatin group (*n* = 40)	Rosuvastatin group (*n* = 40)	*P*-value
CRP (mg/L)	Baseline	47 ± 25	53 ± 23	0.25
	After 3 months	20 ± 10	16 ± 6	0.024^a^
	*P*-value	< 0.001^b^	< 0.001^b^	
MMP9 (ng/L)	Baseline	155 ± 43	144 ± 17	0.15
	After 3 months	130 ± 42	104 ± 33	0.003^a^
	*P*-value	0.001^b^	< 0.001^b^	

*CRP*, C-reactive protein; *MMP9*, matrix metalloproteinase-9. Data are represented as mean ± SD for quantitative normally distributed data. ^a^Statistically significant difference between the two groups. ^b^Statistically significant difference between baseline and after treatment in each group. Unpaired *t*-test and paired *t*-test as appropriate statistically significant difference at *P* < 0.05

The sST2 level decreased by 6.1% after 3 months of treatment as compared to baseline in the rosuvastatin group [27 (20/31) vs. 28 (21/32) ng/ml] while decreased by 2.3% in the atorvastatin group [21 (17/27) vs. 22 (19/28) ng/ml], this may suggest that rosuvastatin slightly reduces sST2 levels more than atorvastatin. Although the median percentage change values showed a reduction in sST2 level in both groups, the difference between the two groups was statistically insignificant **(**Table 4**)**.

**Table 4:** Effect of high-dose atorvastatin and rosuvastatin on sST2 at baseline and after treatment

Parameters		Atorvastatin group (*n* = 40)	Rosuvastatin group (*n* = 40)	*P*-value
sST2 (ng/ml)	Baseline	22 (19/28)	28 (21/32)	0.079
	After 3 months	21 (17/27)	27 (20/31)	0.024^a^
	*P*-value	0.288	0.181	
	Median change (%)	− 2.3 (− 21/13)	− 6.1 (− 17/7)	0.840

*sST2*, soluble suppression of tumorigenicity 2. Data are represented as median (25th/75th percentile) for quantitative non-normally distributed data. ^a^Statistically significant difference between the two groups. Mann–Whitney test and Wilcoxon signed ranks test as appropriate statistically significant difference at *P* < 0.05. Percentage change was calculated as [(post 3 months – baseline)/baseline] × 100

**Fig. 2:**
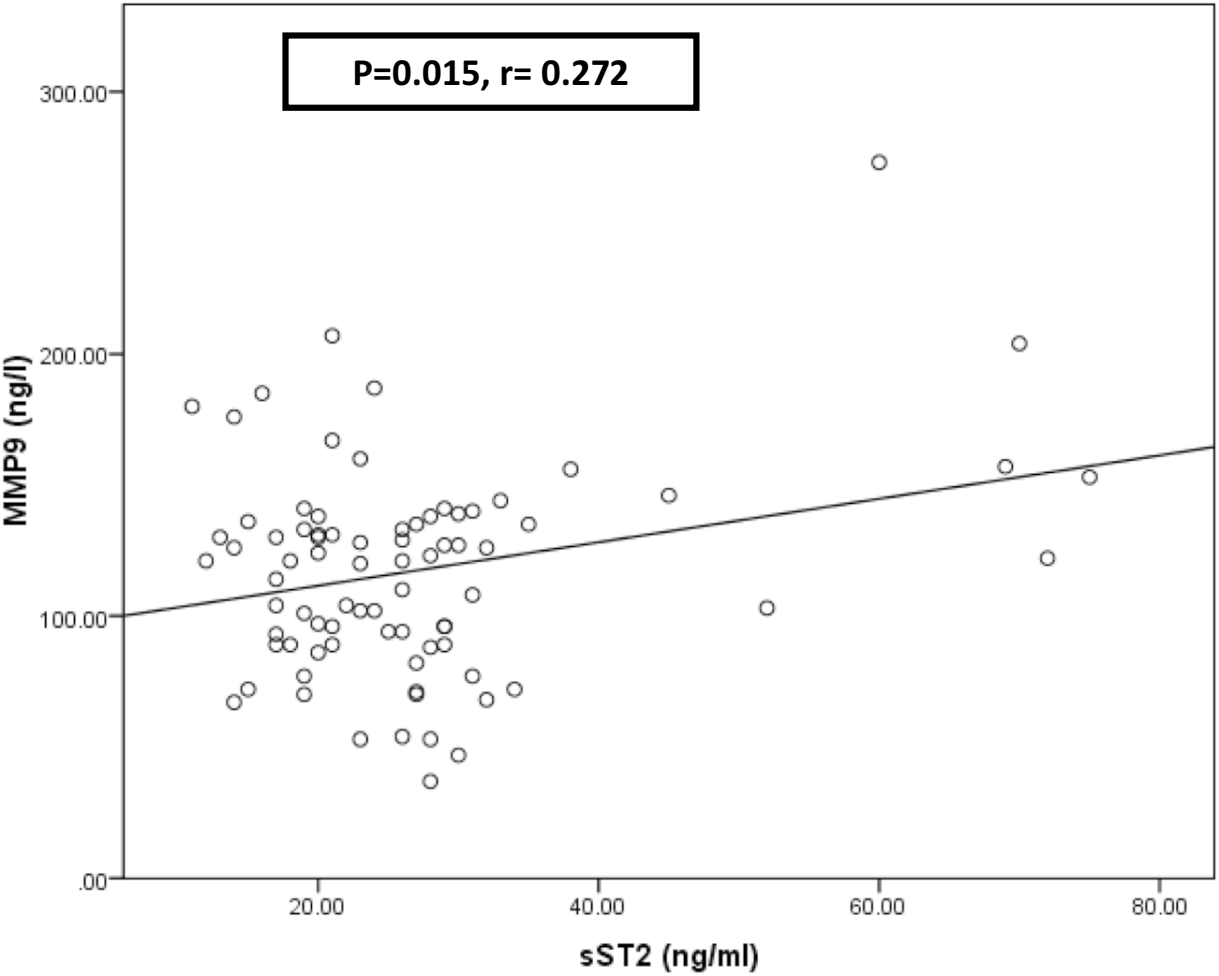
Pearson correlation of Matrix Metalloproteinase-9 (MMP9) and soluble Suppression of Tumorigenicity 2 (sST2). Statistically significant at *P* < 0.05

Pearson correlation was performed, and a significant positive correlation existed between MMP9 and sST2 (*r* = 0.272, *P* = 0.015) **(**Fig. 2**)**.

### Effect of Statins on Echocardiographic Parameters

A statistically significant improvement in LVEF% was observed after 3 months of treatment in both atorvastatin and rosuvastatin groups compared to their corresponding baseline values (*P* < 0.05). However, no significant difference was observed in LV volumes. Post-treatment, there was a significant increase in LVEF% in the rosuvastatin group compared to the atorvastatin group (48.5 ± 9 vs. 43.5 ± 11%, *P* < 0.05). At the same time, LVEDV and LVESV were significantly decreased in the rosuvastatin group compared to the atorvastatin group (101 [81/135] vs. 134 [100/150] ml) (53 [37/75] vs. 73 [52/92] ml) (*P* < 0.05), respectively **(**Table 5**)**.

**Table 5:** Effect of high dose atorvastatin and rosuvastatin on echocardiography parameters at baseline and after treatment

Parameters		Atorvastatin group (*n* = 40)	Rosuvastatin group (*n* = 40)	*P*-value
LVEDV (ml)	Baseline	116 (96/142)	105 (89/141)	0.25
	After 3 months	134 (100/150)	101 (81/135)	0.041^a^
	*P*-value	0.267	0.9	
LVESV (ml)	Baseline	74 (49/88)	59 (47/77)	0.156
	After 3 months	73 (52/92)	53 (37/75)	0.033^a^
	*P*-value	0.762	0.173	
LVEF (%)	Baseline	40.7 ± 10	44.7 ± 9	0.072
	After 3 months	43.5 ± 11	48.5 ± 9	0.029^a^
	*P*-value	0.02^b^	0.001^b^	

*LVEF*, left ventricular ejection fraction; *LVEDV*, left ventricular end-diastolic volume; *LVESV*, left ventricular end-systolic volume. Data are represented as mean ± SD for quantitative normally distributed data median (25th/75th percentile) for quantitative non-normally distributed data. ^a^Statistically significant difference between the two groups. ^b^Statistically significant difference between baseline and after treatment in each group. Unpaired *t*-test, paired *t*-test, Mann–Whitney test, and Wilcoxon signed ranks test, as appropriate statistically significant difference at *P* < 0.05

### Monitoring Adverse Effects

No significant elevation was observed in liver enzymes (ALT and AST) after 3 months of treatment in both atorvastatin and rosuvastatin groups **(**Table 2**)**. In both groups, a few gastrointestinal side effects (constipation and dyspepsia) and headache were observed. In the atorvastatin group, three patients suffered from constipation (7.5%) and headache (7.5%), and two patients suffered from dyspepsia (5%). In the rosuvastatin group, two patients suffered from constipation (5%), and one patient suffered from dyspepsia (2.5%) and headache (2.5%). However, no patients suffered from myalgia in both groups. All adverse effects were well tolerated and did not require statin discontinuation. There was no statistically significant difference in the occurrence of adverse events between the two groups (*P* > 0.05) **(**Table 6**)**.

**Table 6:** Adverse effect profile

Type of ADR	Atorvastatin group (*n* = 40)	Rosuvastatin group (*n* = 40)	*P*-value
Constipation	3 (7.5%)	2 (5%)	0.655
Dyspepsia	2 (5%)	1 (2.5%)	0.564
Headache	3 (7.5%)	1 (2.5%)	0.317
Myalgia	0	0	1

*ADR*, adverse drug reaction. Chi-square as appropriate, statistically significant between groups at *P* < 0.05

## Discussion

STEMI patient’s myocardium is severely ischemic and necrotic [31]. Minimizing the damage in this injured myocardium is essential, so the current management aims to prevent cardiac necrosis, LVR, and subsequent heart failure development [31]. Recent research suggests that statins may also improve LVR by preventing cardiac fibroblast proliferation and the turnover of ECM [6]. Some studies suggested the antihypertensive effect of statins which may be due to their pleotropic effect [32–34]. The additional antihypertensive effect of statin when co-administrated with antihypertensive drugs may contribute to reducing cardiac remodeling [32, 33]. According to ESC guideline recommendations, high doses of rosuvastatin and atorvastatin are the two most widely prescribed statins in clinical settings for STEMI managemen [15]. Nevertheless, it is still controversial as to whether high-dose atorvastatin or rosuvastatin should be prescribed to these patients.

In the current study, high-dose rosuvastatin (20 mg/d) compared with high-dose atorvastatin (40 mg/d) was superior in improving echocardiography parameters, inflammatory, myocardial fibrosis, and hypertrophy biomarkers after 3 months in STEMI patients, suggesting that high-dose rosuvastatin may improve LVR and cardiac function significantly more than high-dose atorvastatin therapy. These results were in line with the findings of other studies. The study by Zhou et al. compared the effect of 20 mg of rosuvastatin to 40 mg of atorvastatin on the inflammatory factors (TNF-α, IL-6, and hs-CRP), and LVR for 3 months in non-STEMI patients demonstrated a significant improvement in echocardiographic parameters in the rosuvastatin group compared to the atorvastatin group [35]. This finding was because rosuvastatin inhibited neurohormonal activation and improved serum inflammatory factors more efficiently than atorvastatin, which plays an essential role in LVR [35]. Hence, rosuvastatin more effectively reduced myocardial injury, fibrosis, and hypertrophy [35]. In another 6-month pilot study by Chitose et al., the effect of 5 mg rosuvastatin versus 10 mg atorvastatin treatment in STEMI patients was investigated [31]. LVEF was improved only by rosuvastatin. Also, the reduction of brain natriuretic peptide (BNP) levels was more significant in the rosuvastatin group [31]. As a result, rosuvastatin appeared to be more effective than atorvastatin for long-term improvement in cardiac function in STEMI patients [31]. 

Using multiple biomarkers by integrating both standard and new biomarkers in patients with post-MI remodeling increases their clinical outcomes’ sensitivity and specificity [6]. Therefore, in the present study, MMP9 and sST2, promising biomarkers for cardiac fibrosis and hypertrophy, and CRP, a conventional biomarker for inflammation [11], were measured in addition to echocardiography. To the best of our knowledge, this is the first randomized study to compare the effect of high doses of atorvastatin or rosuvastatin on STEMI patients’ LVR and prognosis using multiple biomarkers.

CRP is a non-specific inflammatory biomarker and is positively related to remodeling. Clinical studies have shown that high CRP levels following MI were linked to adverse clinical outcomes such as LVR, heart failure development, and death [10, 36]. A study by Khurana et al. undertaken in STEMI patients showed that 20 mg of rosuvastatin significantly decreased the CRP level after 4 weeks of treatment compared to 40 mg of atorvastatin [37]. In other studies, rosuvastatin significantly reduced other inflammatory biomarkers (hs-CRP and ESR) more than atorvastatin in STEMI patients after 4 weeks [38, 39]. These results supported our finding, as a significant reduction in the level of CRP was shown in the rosuvastatin group compared to the atorvastatin group, suggesting that 20 mg of rosuvastatin has a more potent anti-inflammatory effect than 40 mg of atorvastatin in STEMI patients after 3 months.

MMP9 is a sturdy and very early biomarker for left ventricular remodeling in MI patients [40]. MMP9, mainly produced by macrophages, is primarily responsible for degrading and reshaping the dynamic balance of the ECM [13, 22]. So, it is associated with the vulnerability of plaque and LVR after MI. Thus, it is important to predict LVR and the patient’s prognosis [13]. Guo et al. and Luo et al. demonstrated that a high dose of rosuvastatin significantly reduced MMP9 serum level in AMI patients and improved echocardiographic parameters, LVR, and cardiac function [9, 41]. Zhijian et al. demonstrated that high-dose atorvastatin significantly decreased MMP9 levels in AMI patients and improved only LVEF [36]. These previous findings demonstrated that both statins lower MMP9 levels and improve cardiac function; thus, we conducted this study to determine which was more effective. Our results demonstrated that both groups significantly reduced MMP9 levels after 3 months of treatment compared to baseline, but the reduction in the rosuvastatin group was more significant. So, high-dose rosuvastatin may prevent and treat myocardial hypertrophy and fibrosis more efficiently than high-dose atorvastatin.

In patients with MI, sST2 has recently appeared as a biomarker for ventricular fibrosis and remodeling [42, 43]. The elevated sST2 level in these patients was strongly related to increased cardiac fibrosis and post-MI ventricular remodeling development [44]. Furthermore, there was a promising result regarding sST2 predictive value related to heart failure development and mortality for MI patients [11]. Some studies demonstrated that statin reduced the level of sST2 in HIV and multiple myeloma patients [45–47]. Our study has been the first to investigate the impact of high-dose atorvastatin and rosuvastatin on serum sST2 levels in STEMI patients. The level of sST2 was reduced in both groups after 3 months of treatment compared to baseline, but high-dose rosuvastatin may slightly reduce sST2 level more than high-dose atorvastatin. This finding suggested that high-dose rosuvastatin may prevent and treat myocardial hypertrophy and fibrosis more than high-dose atorvastatin. So, further large studies are needed to confirm that statins reduce sST2 levels in patients with STEMI. Also, these results may interest other researchers in understanding whether the reduction in sST2 level by high-dose rosuvastatin contributes to its beneficial effects in improving LVR and cardiac function during the STEMI patients’ management.

Furthermore, Doppler echocardiography was performed to observe the effect of both statins on LVR. A significant improvement in echocardiographic parameters was observed in the rosuvastatin group compared to atorvastatin. This finding may support the superiority of rosuvastatin over atorvastatin in improving LVR and cardiac function. As far as we know, statins possess a pleiotropic effect independent of their lipid-lowering effect [48]. Regarding the lipid profile of the two groups in this study, both drugs showed comparable effects on all lipid parameters. Therefore, the better improvement in LVR observed in the rosuvastatin group may explain that high-dose rosuvastatin has a greater pleiotropic effect than high-dose atorvastatin.

There is no clear explanation for the observed superior effect of rosuvastatin over atorvastatin regarding the outcome in STEMI patients. Possible explanations for rosuvastatin’s superior effect may be its structure and physical properties. First, rosuvastatin was shown to make more bonds with HMG-CoA reductase than other statins, thus having the highest inhibitory potency [49]. Second, the pharmacokinetics of rosuvastatin and atorvastatin were different [50, 51]. Rosuvastatin has a longer elimination half-life of ∼ 19 h than atorvastatin ∼ 14 h, which could explain its superior efficacy and potency [50, 51]. Third, the lipophilic/hydrophilic properties of both statins may affect the outcome of the damaged myocardium after acute STEMI [31]. Lipophilic atorvastatin can enter the lipid membrane of extrahepatic cells, including damaged myocardium, and inhibit mevalonate synthesis, leading to the prevention of isoprenoid intermediates and ubiquinone (coQ10) production, thereby affecting mitochondrial function and slowing down adenosine triphosphate (ATP) generation, possibly affecting myocardium contraction [31, 52]. This deleterious effect may cancel the beneficial impact of atorvastatin in patients with ischemic heart disease. In contrast, the hydrophilic rosuvastatin cannot enter extrahepatic cells, including damaged myocardium, and cannot reach enzymes inside the cells [31, 52]. So, rosuvastatin may have a reduced effect on the HMG-CoA-mevalonic acid pathway in damaged myocardium after STEMI, reducing the impact on the ATP-generating system [31, 52]. These suggest that under acute myocardial ischemia, more cardioprotective effect may be observed with hydrophilic statins than hydrophobic statins, particularly in patients with STEMI [31].

## Limitation

There were some limitations in this study. All included patients were from a single center, and we could not evaluate the long-term prognosis. So, future large-scale, long-term, multicenter studies are needed. Second, the current study has been the first to investigate the impact of high-dose atorvastatin and rosuvastatin on serum sST2 levels in STEMI patients, so, larger, long-term, multicenter studies are needed to confirm that high-dose atorvastatin and rosuvastatin reduce sST2 levels in patients with STEMI.

## Conclusion

Clinicians’ decision-making can be enhanced by understanding which high-intensity statin can better improve prognosis after MI, especially in STEMI patients. By combining all the present study results, patients treated with high dose rosuvastatin were associated with more improvement in echocardiographic parameters and a more significant reduction in inflammatory, fibrosis, and hypertrophy biomarkers, thus improving ventricular remodeling, cardiac function and predicting prognosis better in STEMI patients.

## Supplementary Information

Below is the link to the electronic supplementary material.

Supplementary file1 (XLSX 10 KB)

Supplementary file2 (DOCX 19 KB)

## Data Availability

The data that support the findings of this study are available from the corresponding author upon request.
