# Supplementary material for: Comparative Study Between the Effects of High Doses of Rosuvastatin and Atorvastatin on Ventricular Remodeling in Patients with ST-Segment Elevation Myocardial Infarction
Source: Cardiovasc Drugs Ther. 2024 Sep 12;39(5):1113–23. doi: 10.1007/s10557-024-07621-w (PMC12602584; doi:10.1007/s10557-024-07621-w)
Supplement: Supplementary file 2 — Supplementary file2 (DOCX 19 KB) [file 10557_2024_7621_MOESM2_ESM.docx]

| **Group** | **sST2 level at baseline** | **sST2 level after 3 months of treatment** |
| --- | --- | --- |
| Atorvastatin 40 mg group | 22 | 14 |
| Atorvastatin 40 mg group | 37 | 31 |
| Atorvastatin 40 mg group | 26 | 17 |
| Atorvastatin 40 mg group | 71 | 75 |
| Atorvastatin 40 mg group | 20 | 17 |
| Atorvastatin 40 mg group | 62 | 52 |
| Atorvastatin 40 mg group | 19 | 20 |
| Atorvastatin 40 mg group | 23 | 23 |
| Atorvastatin 40 mg group | 12 | 14 |
| Atorvastatin 40 mg group | 24 | 17 |
| Atorvastatin 40 mg group | 25 | 23 |
| Atorvastatin 40 mg group | 18 | 21 |
| Atorvastatin 40 mg group | 21 | 19 |
| Atorvastatin 40 mg group | 21 | 18 |
| Atorvastatin 40 mg group | 18 | 20 |
| Atorvastatin 40 mg group | 22 | 21 |
| Atorvastatin 40 mg group | 19 | 21 |
| Atorvastatin 40 mg group | 16 | 11 |
| Atorvastatin 40 mg group | 30 | 16 |
| Atorvastatin 40 mg group | 27 | 24 |
| Atorvastatin 40 mg group | 27 | 18 |
| Atorvastatin 40 mg group | 18 | 21 |
| Atorvastatin 40 mg group | 64 | 70 |
| Atorvastatin 40 mg group | 18 | 20 |
| Atorvastatin 40 mg group | 20 | 14 |
| Atorvastatin 40 mg group | 19 | 26 |
| Atorvastatin 40 mg group | 15 | 17 |
| Atorvastatin 40 mg group | 70 | 60 |
| Atorvastatin 40 mg group | 23 | 26 |
| Atorvastatin 40 mg group | 21 | 22 |
| Atorvastatin 40 mg group | 21 | 24 |
| Atorvastatin 40 mg group | 28 | 21 |
| Atorvastatin 40 mg group | 22 | 17 |
| Atorvastatin 40 mg group | 24 | 29 |
| Atorvastatin 40 mg group | 19 | 27 |
| Atorvastatin 40 mg group | 39 | 69 |
| Atorvastatin 40 mg group | 17 | 26 |
| Atorvastatin 40 mg group | 25 | 28 |
| Atorvastatin 40 mg group | 32 | 29 |
| Atorvastatin 40 mg group | 32 | 23 |
| Rosuvastatin 20 mg group | 17 | 26 |
| Rosuvastatin 20 mg group | 19 | 20 |
| Rosuvastatin 20 mg group | 17 | 20 |
| Rosuvastatin 20 mg group | 12 | 12 |
| **Group** | **sST2 level at baseline** | **sST2 level after 3 months of treatment** |
| Rosuvastatin 20 mg group | 28 | 26 |
| Rosuvastatin 20 mg group | 21 | 19 |
| Rosuvastatin 20 mg group | 21 | 15 |
| Rosuvastatin 20 mg group | 34 | 28 |
| Rosuvastatin 20 mg group | 32 | 13 |
| Rosuvastatin 20 mg group | 33 | 31 |
| Rosuvastatin 20 mg group | 51 | 33 |
| Rosuvastatin 20 mg group | 14 | 15 |
| Rosuvastatin 20 mg group | 20 | 34 |
| Rosuvastatin 20 mg group | 24 | 19 |
| Rosuvastatin 20 mg group | 31 | 27 |
| Rosuvastatin 20 mg group | 24 | 30 |
| Rosuvastatin 20 mg group | 28 | 19 |
| Rosuvastatin 20 mg group | 62 | 38 |
| Rosuvastatin 20 mg group | 20 | 30 |
| Rosuvastatin 20 mg group | 32 | 29 |
| Rosuvastatin 20 mg group | 24 | 23 |
| Rosuvastatin 20 mg group | 29 | 31 |
| Rosuvastatin 20 mg group | 32 | 29 |
| Rosuvastatin 20 mg group | 25 | 28 |
| Rosuvastatin 20 mg group | 25 | 23 |
| Rosuvastatin 20 mg group | 27 | 32 |
| Rosuvastatin 20 mg group | 75 | 45 |
| Rosuvastatin 20 mg group | 79 | 72 |
| Rosuvastatin 20 mg group | 26 | 28 |
| Rosuvastatin 20 mg group | 33 | 35 |
| Rosuvastatin 20 mg group | 31 | 19 |
| Rosuvastatin 20 mg group | 34 | 30 |
| Rosuvastatin 20 mg group | 18 | 25 |
| Rosuvastatin 20 mg group | 28 | 28 |
| Rosuvastatin 20 mg group | 28 | 26 |
| Rosuvastatin 20 mg group | 31 | 32 |
| Rosuvastatin 20 mg group | 17 | 27 |
| Rosuvastatin 20 mg group | 37 | 27 |
| Rosuvastatin 20 mg group | 27 | 29 |
| Rosuvastatin 20 mg group | 21 | 20 |
